# Incentivizing Prevention: Community Based Organizations’ Perceptions on Financial Incentives for Recruitment and Retention of Participants in Keep it Up! (KIU!), an eHealth HIV Prevention Intervention

**DOI:** 10.1007/s10461-025-04963-w

**Published:** 2025-11-21

**Authors:** Alithia Zamantakis, Juan Pablo Zapata, Justin D. Smith, Elizabeth C. Danielson, Kathryn Macapagal, Rana Saber, Dennis Li, Nanette Benbow, Brian Mustanski

**Affiliations:** 1 Impact Institute, Northwestern University, 625 N Michigan Ave., Ste. 1400, Chicago, IL 60611, USA; 2 Department of Medical Social Sciences, Northwestern University, 633 N. St. Clair St, Chicago, IL, USA; 3 Department of Surgery, University of Chicago, 5841 S. Maryland Avenue, Chicago, IL, USA; 4 Department of Population Health Sciences, Spencer Fox Eccles School of Medicine, University of Utah, Salt Lake City, UT, USA

**Keywords:** Financial incentives, HIV prevention, Community-based organizations, Digital intervention, Recruitment and retention

## Abstract

While financial incentivization has been found to significantly increase HIV retesting, receipt of HIV test results, and viral suppression, little research has examined community-based organizations (CBOs) perspectives on implementing financial incentives. We interviewed 22 CBOs related to implementing financial incentives for client engagement in a digital HIV prevention intervention, Keep It Up! (KIU!). KIU! was designed with and for young men who have sex with men (YMSM) who tested HIV-negative to maintain their negative status by reducing risk and enhancing protection. Most CBOs changed their incentive amounts and structures across two years of implementation to attempt to increase recruitment and retention. CBOs originally proposed a mean of $39 incentive in their applications for funding. One year later, the average incentive increased to $40, and then $68 the next year. Most CBO staff felt incentives may be effective in increasing recruitment, retention, and engagement. They highlighted the role of incentives in alleviating social determinants of health. Nearly a third of staff, though, felt the size of incentives may not be high enough. Further, these staff highlighted that incentives may be most helpful in recruiting participants but less beneficial for retention and engagement. Our analysis is among the first to examine CBO staff perceptions of implementing financial incentives for digital HIV prevention interventions in the U.S. Future research is needed to assess the effectiveness of financial incentivization of digital HIV prevention interventions and to identify the amount an incentive should be to increase recruitment and retention.

**Trial Registration:**

NCT03896776.

## Resumen

Resumen Si bien se ha descubierto que los incentivos financieros aumentan significativamente las pruebas de VIH, la recepción de los resultados de las pruebas y la supresión viral, pocas investigaciones han examinado las perspectivas de las organizaciones comunitarias (OC) sobre la implementación de incentivos financieros. Entrevistamos a 22 OCs con respeto a la implementación de incentivos financieros para la participación de los clientes en una intervención digital de prevención del VIH, Keep It Up! (KIU!). KIU! fue diseñado con y para YMSM que dieron negativo en la prueba de VIH para mantener su estado negativo al reducir el riesgo y mejorar la protección. La mayoría de las OBC cambiaron los montos y las estructuras de sus incentivos a lo largo de los dos años de implementación para intentar aumentar el reclutamiento y la retención. Las OBC originalmente propusieron un incentivo promedio de $39 en sus solicitudes de financiamiento. Un año después, el incentivo promedio aumentó a $40 y luego a $68 al año siguiente. La mayoría del personal de las organizaciones comunitarias (OBC) consideró que los incentivos podrían ser eficaces para aumentar el reclutamiento, la retención y la participación. Destacaron su papel en la mitigación de los determinantes sociales de la salud. Sin embargo, casi un tercio del personal consideró que la magnitud de los incentivos podría ser insuficiente. Además, este personal destacó que los incentivos podrían ser más útiles para reclutar participantes, pero menos beneficiosos para la retención y la participación. Nuestro análisis es uno de los primeros en examinar las percepciones del personal de las OBC sobre la implementación de incentivos financieros para intervenciones digitales de prevención del VIH en EE. UU. Se necesitan investigaciones futuras para evaluar la eficacia de la incentivación financiera de las intervenciones digitales de prevención del VIH e identificar el monto que debería tener un incentivo para aumentar el reclutamiento y la retención.

## Introduction

Health promotion research often provides financial incentives for participation. But, the question remains: are these same incentives necessary when the prevention intervention moves out of the lab and into implementation? Furthermore, is it ethical or sustainable to offer financial rewards for engagement in a prevention program, potentially overshadowing the importance of intrinsic motivation for self-care? Most of these questions have been examined in research on contingency management (CM), or the provision of financial incentives for engaging in set behaviors (e.g., providing a cash payment for achieving viral suppression and then increasing that cash payment for each additional month that the patient maintains viral suppression [1]). Financial incentives range from the provision of cash, gift cards, or other financial payments for completion of intervention milestones to CM for engaging in set behaviors.

Recent studies detail the mixed effect of financial incentives on health outcomes. Financial incentivization in randomized clinical trials focused on weight loss interventions [2], increasing physical activity [3], vaccine uptake [4], and substance use treatment [5–8] has led to significant increases in engagement in healthy behaviors and reduced engagement in unhealthy behaviors. However, other trials focused on increasing glycemic control and weight reduction [9, 10] have found no significant effect of financial incentives on intended outcomes. Within the field of HIV/AIDS research, financial incentives have been found to significantly increase HIV testing [11], HIV retesting [12], receipt of HIV test results [13], and viral suppression [14–18]. Mixed findings have been reported regarding financial incentives on condom use, with some studies finding that financial incentives increase condom use among cisgender men [19] and others finding no significant effect [20].

One explanation for these mixed findings is financial incentive variation. For example, a study examining the impact of financial incentives on reducing HIV, HSV-2, and Syphilis incidence in Tanzania found that a $20 incentive significantly reduced incidence; however, the $10 incentive had no impact [21]. Similarly, another study providing $0–32 based on a random lottery to cisgender men and women in rural Malawi found that the highest incentive significantly reduced engagement in vaginal sex for cisgender women, but cisgender men receiving an incentive were actually more likely to engage in vaginal sex [19]. In this vein, several studies have found incentive-influenced reductions in STIs [22] and engagement in vaginal sex [19], as well as uptake of COVID vaccines, to vary by race and gender, with young men and women, as well as Black individuals and white individuals, responding differently to incentives [23]. Thus, variation in findings may also be attributable to racialized or gendered differences in populations, size of incentive, and the economic status of a nation. In the U.S., research on cocaine use treatment has found that a $240-prize-based CM intervention significantly reduced cocaine use and had durable effects post-intervention, while an $80 comparison did not have a significant effect on substance use [5]. Other studies have found voucher-based methods to significantly reduce cocaine use and/or increase engagement and retention in substance use treatment; however, voucher-based CM interventions tend to have significantly larger costs (e.g., approximately $1,000 per client across 3 months; [24]). While these amounts for CM interventions are quite large, the actual amount earned by patients is often significantly lower due to real-world circumstances (e.g., missing a session, not meeting a milestone). Incentive amounts may also ultimately be lowered or altered by institutional review boards (IRBs) to ensure that incentives are not coercive (i.e., overt threat of harm) or inserting undue influence (i.e., excessive amount that compromises a reasonable person ability to evaluate the risks) into the trial [25]. IRBs tend to be more cautious in approving incentive rates; however, researchers have highlighted that, while incentives cannot by definition be coercive, efforts should be taken to make sure they do not represent undue influence [25].

With the rise in incentives for engagement in care, some public health agencies are worried about consequences and the challenges of scaling and sustaining financial incentives within traditional care settings [26, 27]. These consequences include reduced attention to non-incentivized clinical activities, a decline in provider professionalism, lower job morale and satisfaction, increased health disparities, and the reduction of intrinsic motivation [28–31]. An implementation science approach can provide insights on how these incentives can be enhanced in real time, their mechanisms of change, and the contexts in which they operate effectively [32]. For instance, a recent mixed-method implementation study in Louisiana evaluated both the implementation and clinical outcomes associated with conditional financial incentives for people living with HIV [33]. The study found that financial incentives positively supported engagement and retention in HIV care. However, implementation partners from participating clinics expressed a preference for lower incentive amounts during the initial planning phase of the trial, citing concerns about the sustainability of larger incentives. Consequently, the incentives were adjusted accordingly (up to $140 in total per participant). While they found financial incentives increased engagement in care, there was not an association between incentives and viral suppression. Further research is needed incorporating implementation science frameworks to identify preferences for these incentives in HIV prevention, to explore the facilitators and barriers to their implementation, and to evaluate the effectiveness of financial incentives.

There is, additionally, an increased need to understand the process of implementing financial incentives from the community and organizational perspective. Limited research on the impact of financial incentives has been carried out within HIV implementation research, or the scientific study of system-level changes to increase reach, effectiveness, adoption, sustainment, and equitable delivery of evidence-based interventions (EBIs; [34]). HIV clinic staff, including clinicians, HIV testers, patient navigators, and administrative staff, are often involved in HIV implementation research, yet less research has attended to their perspectives on the implementation of incentives.

Research with HIV clinic staff on barriers and facilitators to implementing financial incentives has found that providers worry about the cost of incentives amid limited available resources, about the sustainability of financial incentives (both in terms of cost and on long-term impact on patients), that patients will falsify information to receive money, and that it is inappropriate to incentivize individuals to care for their health [35–37]. However, once financial incentives have been implemented, clinicians report increased motivation, reduced stigma, and improved communication and rapport with patients [37]. Further, patients have expressed surprise at incentives being offered and were encouraged to engage in set behaviors (e.g., ART initiation) in response [36]. Of note, the timing of incentives may particularly matter for linkage to HIV care, as prior research has detailed the discomfort and anger of patients being offered an incentive upon first receiving an HIV diagnosis [38]. Further research is needed to better understand the efficacy of financial incentives for HIV prevention. While there are likely similarities in how financial incentives may impact HIV prevention and HIV treatment, there are differences researchers need to consider, as well. For example, while people with HIV need to take ART their entire lives, not all individuals need to take pre-exposure prophylaxis (PrEP) for HIV prevention for the entirety of their lives; thus, considerations of durability may need to be different for HIV prevention than for HIV treatment. It is possible, as well, that financial incentives, depending on their size, could mitigate the role of poverty as a critical driver of individuals acquiring HIV [39, 40].

In this paper, we examine community-based organization (CBO) staff perceptions on financial incentives for recruitment, retention, and engagement in a digital health intervention (DHI) for HIV prevention in twenty-two CBOs. Here, we use “recruitment” to refer to the process of enrolling an individual in an intervention. This process requires attention to the role and perspectives of CBO staff and leadership, as it is these individuals who are funded to enroll their (potential) clients into myriad interventions [41]. We use “engagement” to refer to clients actively engaging in “the intervention as intended by the developers or prescribers of the intervention” [42]. Finally, we use “retention” to refer to the process of keeping clients or patients engaged in the core activities of the intervention.

## Methods

This manuscript reports implementation data collected in the context of a type III effectiveness–implementation hybrid study [43, 44] that compares two delivery approaches for a digital HIV prevention EBI: direct-to-consumer and CBO-based implementation. The study protocol is described elsewhere [45]. In this manuscript, we focus on the CBO-based implementation arm. Twenty-two CBOs were selected through a request for proposal (RFP) process like that used by HIV prevention funders [46]. Thirteen CBOs launched implementation in Fall of 2019, seven in Summer of 2020, and two in Fall of 2020.

### Intervention

Keep It Up! (KIU!) is a Centers for Disease Control & Prevention (CDC) designated best-evidence DHI currently in its third iteration. Previous iterations of KIU! are discussed elsewhere [47–50]. KIU! was designed with and for YMSM who tested HIV-negative to “keep it up” and maintain their negative status by reducing risk and enhancing protection. KIU! includes three modules focused on HIV prevention and risk negotiation content. These modules are followed by two “booster” sessions 3 and 6 weeks after completion of the main intervention sessions. Booster sessions include encouragement of regular HIV testing, condom use, and PrEP uptake. The content within KIU! is guided by the information-motivation-behavioral skills model of HIV risk behavior change, with each module engaging participants in particular scenarios and guiding them through behavior choices, the effects of behavioral choices, and asking them to set goals as to their own behaviors [47]. While the intervention does not have PrEP and HIV testing delivery embedded within it, it does provide participants with tools to geolocate sources of PrEP and HIV testing delivery. As noted below, CBOs implementing KIU! either directly offered PrEP and HIV testing or referred out for PrEP. The current study, KIU! 3.0, was implemented in twenty-two community-based organizations (CBOs) located in CDC-priority areas with high HIV rates.

KIU! staff provided virtual training to CBOs on the intervention, capacity building support on how best to integrate it into routine HIV testing, and monthly technical assistance. As part of the training, CBO staff completed the KIU! intervention to best describe it to clients during recruitment. CBO staff also received information about project logistics (e.g., how to use an online administrative dashboard to track recruitment, engagement, and retention, perform project reporting) and time to ask any questions related to implementation within their organizations. As effectiveness of KIU! has been previously demonstrated on behavioral (e.g., condomless anal sex) and biomedical outcomes (e.g., STI incidence), one of the main aims of the KIU! 3.0 trial was to understand how best to scale up implementation of KIU! in CBO settings, with a secondary aim of further establishing effectiveness in the context of broad implementation [47, 49].

### Setting

The twenty-two CBOs implementing KIU! were distributed across all regions of the United States (U.S.), a high-income nation, including one region outside the continental U.S. CBOs had provided HIV services for an average of 21.8 years (IQR: 3–52). Thirteen (59.1%) had previously delivered other CDC-designated evidence-based interventions, including Mpowerment and VOICES/VOCES. Their budgets for KIU! implementation on average was $42,179.5 (IQR: $20,000–68,262). All CBOs had experience delivering HIV testing and either had on-site PrEP services or referred out for PrEP delivery. Among the YMSM that accessed HIV and STI services from these CBOs between 2016 and 2019 (the years immediately prior to KIU! implementation), 43% were white, 22% Black, 10% Asian, 9% multiracial, and 8% American Indian/Alaskan Native; 68% of clients were non-Hispanic/Latino, and 28% were Hispanic/Latino [51].

### Data Collection

KIU! research staff trained in qualitative methods (including authors az, JPZ, and KM) conducted semi-structured, in-depth interviews with two staff from each CBO who were designated by the CBO as implementers of KIU!. Research staff conducting the interviews were not involved in other communication with or support for CBO staff, maintaining a level of distance from those research staff they actively engaged with as part of the implementation process. This was made clear to interviewees to ensure a level of comfort in participating and openly sharing their thoughts. Implementer roles varied across CBOs, with staff holding managerial positions, HIV testing positions, and external communications (i.e., social media, marketing) positions (Table 1). Interviews were based on the original Consolidated Framework for Implementation Research (CFIR; [52]) and conducted between March 2021 and February 2022, when projects were at a mid-point in implementation. Interviews lasted an average of one hour and fifteen minutes (range: 54 min to two hours). We aimed to conduct two interviews per CBO. However, only one staff member participated in interviews from nine of the CBOs, and five CBOs did not participate, resulting in a total of twenty-five interviews. Of the five CBOs who did not participate, their implementation staff did not consent to an interview. CBOs with only one staff member participating had a second staff member who did not consent to participate, resulting in some CBO staff reporting that they had more pressing matters to attend to in terms of their clients’ needs and experiences than participating in additional components of the research study; and 3) there was a moderate level of turnover across CBOs, with an average of 2 staff per CBO involved in the implementation of KIU!.

Interviews were conducted in English with English-speaking participants and occurred over Zoom, a video-conferencing software. The interviews were recorded and automatically transcribed by Zoom. Research staff and investigators (including author az) edited transcriptions to ensure consistency with the audio recording, deidentified transcripts, and uploaded transcripts to MaxQDA for qualitative coding [53]. CBOs also provided near-yearly updates on their incentive rates and allocations.

### Data Analysis

Interview transcripts were coded by three master’s- and doctorate-level researchers and one bachelor’s-level researcher who was trained by the lead author (including authors az and JPZ), using CFIR 1.0 constructs as codes. While CFIR 2.0 [54] came out after the interviews, we chose to retain CFIR 1.0 constructs to match codes with interview questions. We also added an additional code, “incentives,” to capture staff perception on use of incentives across implementation of KIU!. This code was applied to responses to the following questions: (1) how have participant incentives changed from the original plan, if at all; (2) how have participants responded to incentives; and (3) do you perceive incentives to participants as having a role in increasing participant recruitment, retention, and/or engagement? The four researchers double-coded transcripts until they reached intercoder reliability of κ ≥ 0.7. Author az conducted a “codebook approach,” deductive thematic analysis in the following steps: (1) sorting through coded excerpts, comparing and sifting to identify themes using a pattern-based approach to thematic analysis; and (2) sorting through themes to identify their interrelations, ensure mutual exclusivity of themes, and reduce themes to the minimum number needed to fully describe the data (thematic consolidation) [55–57]. Due to CBO staff turnover, we were not able to conduct member checking with interview participants.

We additionally performed descriptive analyses of total incentives across CBOs as well as the average amount of incentives distributed for recruitment, for engagement in core activities, or for retention through the completion of the intervention. We compared the average totals and distributions between what was proposed in responses to request for proposals, what was provided one year into implementation, and what was provided near the end of implementation (i.e., near the end of year 2).

### Positionality

The data collection team included three cisgender individuals and one transgender individual – two of whom were LGBTQ and two of whom were heterosexual. The data collection team was 50% people of color (Asian American and Latino) and 50% white and included three doctoral-level researchers and one master’s-level researcher. The data analysis team included two cisgender individuals and two transgender/nonbinary individuals – three of whom were LGBTQ and one of whom was heterosexual. The data analysis team consisted of three white individuals and one person of color and included two doctoral-level researchers, one master’s-level researcher, and one bachelor’s-level researcher. Our authorship team includes two master’s-level investigators and six doctoral-level investigators and consists of four white individuals, three Latinos, one Asian American individual, and one Arab American individual. All but one of the authors are cisgender.

### Ethics Approval

All procedures performed in studies involving human participants were in accordance with the ethical standards of the institutional and/or national research committee and with the 1964 Helsinki Declaration and its later amendments or comparable ethical standards. The trial was approved by the IRB at Northwestern University (STU00207476). All participants were informed of the aims of the study and of data protection; all participants consented to participate in our trial.

## Results

### CBO Characteristics

Twenty-five staff from twenty-two CBOs participated in qualitative interviews. CBOs had an average of 29 full-time staff members (range = 1–106) and an average of 22 years serving YMSM. Thirteen CBOs (59%) had prior experience implementing HIV EBIs, including Mpowerment [58], SISTA [59], and VOICE/VOCES [60]. However, only six CBOs had previously or at the time of the trial received direct funding from the CDC.

### Thematic Analysis Results

CBO staff varied regarding their perception of whether financial incentives are effective in increasing recruitment and retention. Most staff felt incentives have a positive effect on either recruitment or retention, with only two staff unsure of whether incentives have any benefit. In analyzing staff responses on incentives, six categories of responses emerged: (1) incentives are effective; (2) incentives may not be enough of a motivator for recruitment, retention, or engagement; (3) incentives are beneficial for recruitment but not engagement and retention; (4) incentives need to be substantially higher in dollar amount; (5) non-financial incentives should be considered in addition to financial incentives; and (6) lessons learned in administering incentives.

### Incentives are Effective

Eleven staff across different CBOs perceived incentives to be effective for recruitment. Eight of these staff felt that incentives motivated participants to enroll in KIU!. One staff member explained that when they speak to participants about KIU!, more participants raise their excitement about the incentives than their excitement about the intervention or what they learn. Another staff member responded:

In my observation, it does encourage people to be in the program and remain connected to the program, because they at least get something out of it. They’re getting free testing, they’re getting help, but when you actually monetize something with like a gift card then it looks much more attractive to participants.

Two staff also felt that incentives may have been particularly effective due to the COVID-19 pandemic. With mass layoffs during the height of the pandemic, particularly for Black, Latine, and other people of color [61], many participants may have been searching for other ways to earn additional money. For example, one staff member explained:

Some people do need those incentives during this time. We do Amazon gift cards and virtual gift cards, so who doesn’t shop in Amazon right or who doesn’t need a little extra bit of money during these times?

Another staff member highlighted the height of the pandemic as resulting in people feeling “like their time is even more precious,” leaving people less likely to participate in anything without an immediate benefit. To this staff member, “the incentive makes it a little bit more enticing to participate.” Finally, one other staff member felt that incentives were particularly effective for low-income clients. They noted that a large percentage of their clients do not have income or access to other financial resources. KIU! offered an opportunity to learn how to promote healthy behaviors and to earn extra cash “to survive.”

### Incentives May Not be Enough of a Motivator

Some staff (*n* = 8) felt that incentives may not be enough to motivate their clients to enroll in, engage with, and complete KIU!. Two staff felt that incentives may not be enough to counteract social determinants of health (SDOH) of their clients. One CBO primarily recruited within substance use treatment facilities and homeless shelters, in addition to street outreach. They discussed the difficulty of recruiting individuals to enroll in KIU!, explaining:

Most of the places I would say aren’t conducive for Keep It Up! as far as the people that we’re coming in contact with, because they are sheltered. And so having them complete that program, even with the money as an incentive, isn’t enough for that. The money is not enough. It’s just, their lives are probably a little bit more difficult than to have to keep up with something where they had to watch videos or answer questions.

While this staff member focused on those in treatment facilities and homeless shelters, the other staff member highlighted the difficulty of recruiting anyone during the height of the COVID-19 pandemic due to heightened precarity and financial stress. The incentive, while additional money for one to earn, was not enough in this staff member’s perspective to overcome those barriers.

Two other staff wondered whether incentives were enough but did not have a reason as to why. They did not view incentives as ineffective, but they were not confident that incentives were useful. Finally, three other staff members felt that incentives were not enough to motivate participants alone. However, they explained that financial incentives were necessary in conjunction with participants viewing KIU! as beneficial or of interest. One of these staff members noted:

If the first thing I mention is money, a lot of them are interested, but then when I say, well, you just got to watch a video, has a couple questions, [they respond,] ‘Oh no, I’m not doing all that.’

This staff member highlighted that, if their participants are not interested in KIU!, the incentive becomes less of a motivating factor. Whereas other staff highlighted that individuals who are highly interested in KIU! would participate regardless of the incentives, with one staff member stating:

I’ve noticed that a lot of people have actually completed KIU! and completely forgot about incentives. People who are genuinely willing to do it are going to sit, do it, and enjoy it.

### Incentives Work for Recruitment

Five staff members perceived incentives were effective at increasing recruitment but not engagement or retention. Incentives were an initial way to increase interest in enrolling, but then participants stopped participating and did not complete the intervention. Some staff members argued this may be due to the distribution of incentives across months, rather than a large incentive at each time point. One staff member explained:

Incentives work when you have something that’s significant in that moment. I don’t think they’re, you know, thinking like, ‘Oh, I can’t wait to get 40 more dollars in six months.’ It’s not. Like nobody cares.

This staff member’s perspective was shared by another who found that their clients were excited to complete the baseline and main episode components of KIU!, as they could earn a large amount in one day, but they would not continue to complete remaining episodes. The staff member said:

I tell folks, you know, you will have three episodes available to you right away. So, you can make 60 bucks today, if you want. And so, I think that’s been a really good motivator to get people signed up. Keeping them retained is another thing.

Other staff highlighted that, while clients were interested in enrolling in KIU! and earn the baseline incentive, once it was explained to them what participation entailed, they lost interest. Clients were willing to participate in a brief activity for the incentive, but they were not interested in watching multiple episodes, responding to questions, setting goals, and other activities embedded within KIU!.

### Incentives Need to be Substantially Higher

CBO staff also highlighted that incentives for KIU! needed to substantially increase to be effective in recruiting and retaining participants. Three staff felt that the incentive was too low for the time it takes to complete KIU!. One of these staff discussed the difficulties they were experiencing in recruitment and retention:

Maybe it’s just because it’s time consuming. Or maybe they think that it’s, like, low incentives, because our agency, we provide a $45 incentive in Amazon gift cards. I know others provide more, but that’s what we provide.

This staff member and one other felt that incentives needed to be $80 or more to entice participation. However, the other staff member who also felt incentives were too low, worked at a CBO providing $100 in incentives across the intervention, yet they, too, felt that the amount was too low for an intervention that spanned multiple months of participation. While one of these staff members lived in a city with a cost of living 21% higher than the national average, the other two lived in cities with costs of living lower than the national average.

Four other staff members felt that a large total incentive distributed in smaller amounts across milestones was not as enticing as a large incentive at each milestone. One staff member explained:

When I explain to them and tell them about how fun it is and how interactive it is and that they can receive incentives from doing it, they they’re receptive, but they’re gonna tell you, “I’m not going to do it.” They’re telling me straight up, “I know this is a benefit for me. I know I’m going to receive money, but I don’t have time for this.” Because it’s not like for each episode they’re getting $100, so it’s not like, “Ooh-whee!”

Others explained, as well, that what would be preferable would be to earn $100 within a week or per episode rather than $100 total across several weeks. The same amount can be more attractive to participants in a shorter time, or staff argued, the amount per episode must increase to match the extended length of time for which participants must engage in the intervention.

### Non-financial Incentives

In addition to providing financial incentives, some CBOs offered non-financial incentives (i.e., non-monetary compensation). This included free STI/HIV testing, lubricant, t-shirts, hand fans, earphones, and gift baskets. In total, five CBOs provided non-financial incentives. The CBO that provided a gift basket as compensation decided to do so as a raffle during the height of the COVID-19 pandemic. The main implementation deliverer noticed that as job loss, stay at home measures, and financial insecurity increased, they needed to do more to incentivize participation:

Once I got that okay and [the raffle] approved with my director and supervisor of finance, I purchased two books, one was a photography book about LGBT pride and then the other was a cooking book for singles. Then I also included a cooking apron as well as a pair of socks—that’s something homeless people constantly need. We added a journal, packs of condoms, multiple lubes. I added some butt plugs, a dildo, and then like cock rings. It turned out really well! The person who won the gift basket was really looking forward to it.

Raffles functioned to incentivize more recruitment and retention for CBOs. However, sometimes confusion arose around non-financial incentives. One CBO allowed participants to complete intervention episodes on an iPad at their organization to accommodate those without devices or Wi-Fi. Some participants misunderstood this to mean the CBO offered iPads as incentives and expected to receive one.

One CBO offered free earphones in addition to a financial incentive. They explained:

Last year we had a donation of earphones, and they came in at the right time, because I was just like, alright, well they’re going to be watching videos, here’s an incentive today. You get a pair of earphones by signing up for KIU! and, of course, we only did that, as far as we could until they ran out.

Across CBOs that provided non-financial incentives, they aligned these incentives with the needs and wants of their target population. The CBO that provided free STI/HIV testing had been doing so for many years and built a client base that sought this out. Others aligned the incentive with the intervention, providing, for example, earphones for participants to use for KIU!. Finally, some CBOs had to change plans due to COVID-19. One CBO that originally planned to provide t-shirts and bags as an incentive had to switch to only providing virtual gift cards to the inability to have in-person contact with participants when stay-at-home measures were in place.

### Changes to Incentive Structure

**Fig. 1:**
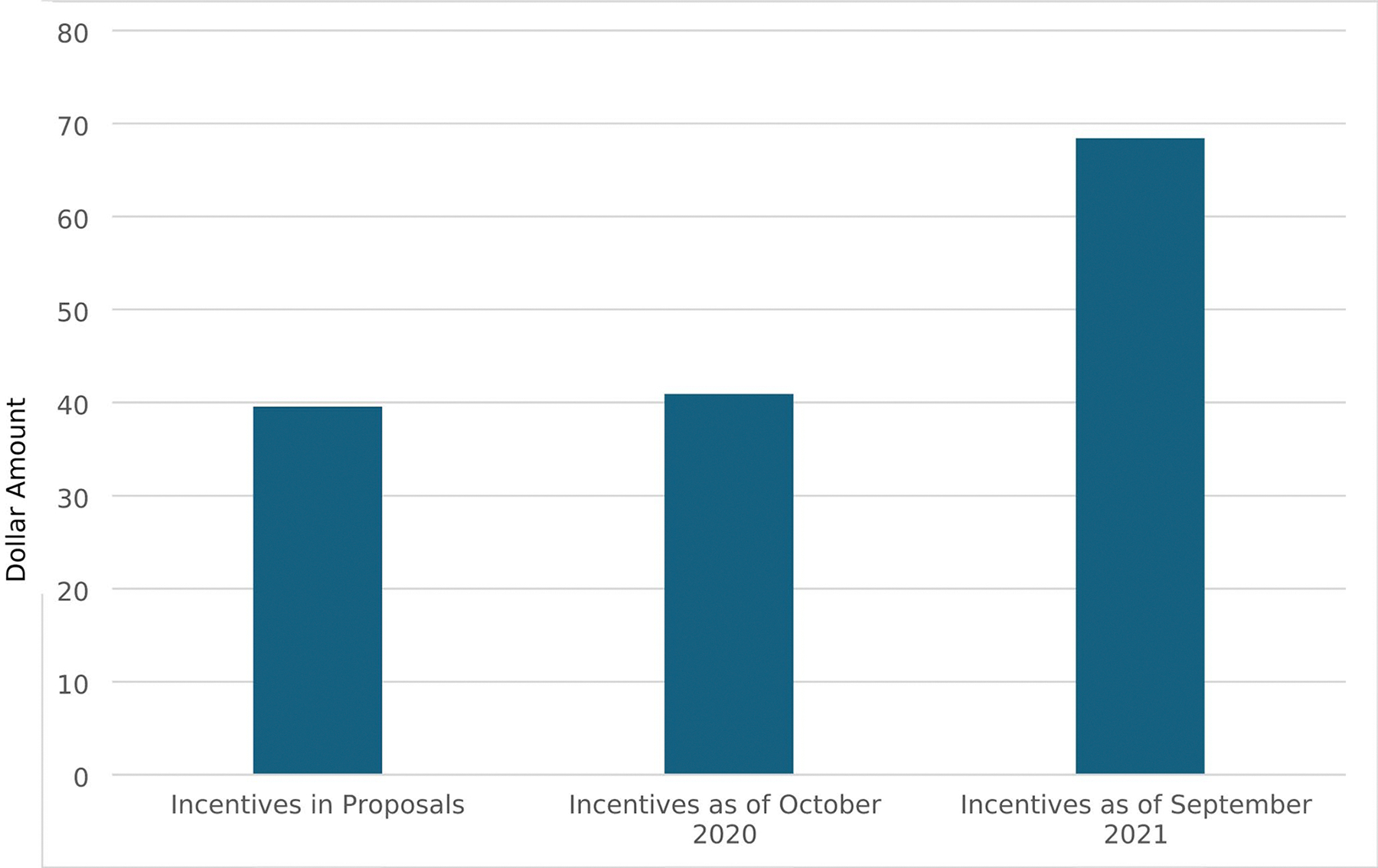
Average incentive amount across implementation. Figure details the average incentive CBOs proposed to provide, the average they did provide as of October 2020, and the average they did provide as of September 2021

**Fig. 2:**
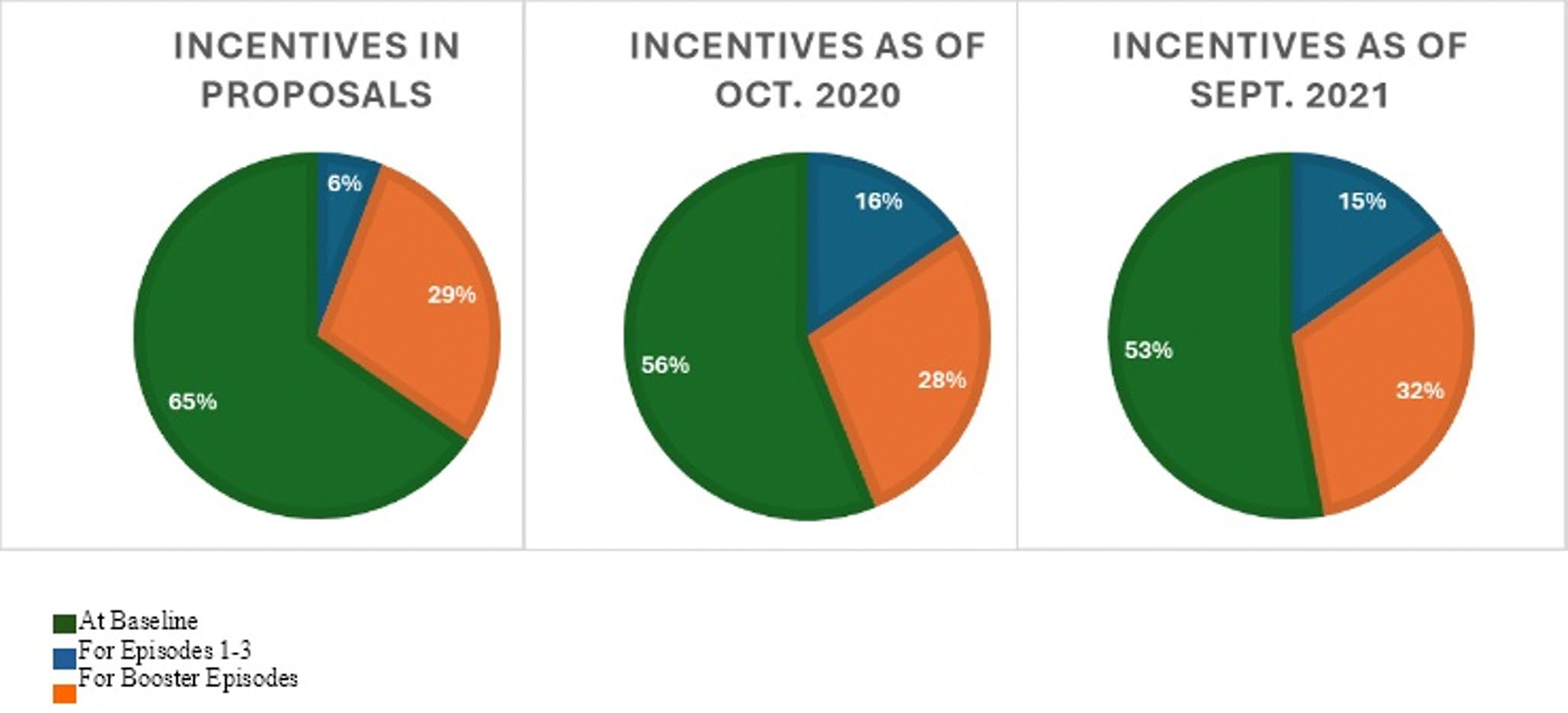
Incentives by intervention component across implementation

Across implementation, CBOs increased and decreased their total incentives and redistributed the amounts given at different milestones within the intervention. In requests for proposals, CBOs identified incentive amounts they proposed to offer if funded. Amounts were distributed by milestone. Milestones included completion of baseline (i.e., completion of eligibility screener, baseline survey, and baseline STI testing), the main intervention episodes (i.e., episodes 1–3, provide the core education content, goal setting, and behavioral reflection), and booster episodes (i.e., two additional episodes that provided nuanced content on pleasure and protection and reinforced content, goals, and reflection from the main episodes). Total incentives included within proposals for funding averaged $39 (range = 0–100; Fig. 1). CBOs proposed to allocate 65% of their total incentive to baseline, 6% to main intervention episodes, and 29% to the booster episodes (Fig. 2).

CBOs reported their incentives again in October 2020, about a year after implementation began. Total incentives at this point averaged $40 (range = 0–150), reflecting only a minimal average increase from the proposed incentives. However, distributions of incentives by milestone exhibited greater change. CBOs allocated, on average, 56% of the total incentive to baseline (a 14% decrease), 16% to main intervention episodes (a 167% increase), and 28% to the booster episodes (a 3% decrease).

Final reports of incentives were collected in September of 2021, almost one year later. Total incentives increased greatly, with the average across CBOs rising to $68 (range = 0–175). CBOs only made slight changes, on average, to distributions of incentives by milestones. CBOs allocated 53% to baseline (a 5% decrease), 15% to main intervention episodes (a 6% decrease), and 32% to booster episodes (a 14% increase). Table 2 reports total incentives per CBO across these three time points across implementation.

CBO staff spoke in interviews about reasons for changing their incentives over time. Six CBOs increased incentives during the two years of implementation, because they were not meeting their goals for enrollment. Another three increased incentives to make KIU! more appealing to participants. When asked about what they might change about their recruitment approach, one staff member explained:

I guess my only thing would be to increase incentive amounts to kind of make it a little bit more appealing and help reach more people.

One other CBO increased their incentive, but they did so only because of the structure of KIU! changed. Mid-implementation due to COVID challenges, the timeline of KIU! module delivery was changed from an original plan of 8-hour breaks between modules 1, 2, and 3, followed by 3-month breaks between module 3 and 4, and module 4 and 5. The 8-hour breaks were removed, and the two 3-month breaks were reduced to 6-week breaks. With the change, the CBO decided to increase the amount of the incentive given at baseline since participants would complete three modules at once. Other CBOs changed the structure of the incentive to be staggered across incentive milestones. For example, one CBO provided $5 to enroll in KIU!, followed by $10 to complete the first three episodes, and then an additional $20 after completing the booster episodes. One CBO began with a non-staggered incentive structure but shifted mid-way. Related to this change, the staff member said:

I really feel strongly on how I charted everything out, on how I spaced everything out, and I think it would be more affordable, as far as just the funding.

Six of the ten CBOs who increased their incentive amounts overlapped with CBO staff who perceived incentives as effective. The remaining four did not comment on the perceived effect of increasing incentives.

## Discussion

This manuscript is among the first to examine CBO staff perceptions of implementing financial incentives for a digital HIV prevention intervention in the U.S. While a great deal of attention has been paid to financial incentivization for HIV prevention and treatment outside the U.S., much less has attended to its role domestically. While prior research has found providers to be hesitant to incentivize healthy behavior [36, 37], some CBO staff implementing KIU! felt that incentives may be helpful for recruiting clients into HIV prevention interventions and may alleviate some SDOH (e.g., poverty, homelessness) for participants who otherwise may not prioritize health interventions. However, some CBO staff also highlighted that the incentive amount was not enough to positively affect recruitment, retention, or engagement or alleviate SDOH. Interestingly, while CBO staff noted this, they simultaneously did not drastically increase incentives for engagement or retention. CBOs initially proposed to allocate 29% of incentives for retention and in the first year of implementation allocated 28% of incentives for retention on average. By the second year of implementation, this number only increased to 32% on average. While incentives for the completion of modules between baseline and booster episodes increased by 9% on average, 53% of incentives, on average, remained allocated to recruitment. There seemed to be a disconnect between CBO staff finding it difficult to engage and retain YMSM yet not drastically shifting incentives to better attend to this. For some CBOs, this may have been due to administrative barriers, as some CBO staff reported a difficulty in altering incentive amounts due to resistance from financial administrators. It is important to reiterate that the research study team did not encourage any particular use of incentives. CBOs were free to use incentives or not and to allocate them as desired, based on their own experience delivering other interventions.

Further, the highest incentive any CBO provided was $175. Only one did so with another seven providing $100–125 across intervention modules. As noted in the introduction, prior research on CM interventions suggests that financial incentives need to be as high as $250–450 if randomly distributed, or up to $1,000 if not randomly distributed, to have a positive effect [5, 24]. This work, though, has been focused on CM for substance use treatment and other non-HIV prevention interventions. On average, CBOs provided less than $70 across intervention modules–significantly lower than what has been found to be effective in fields using CM. Additionally, the low(er) amounts provided as incentives by CBOs for KIU! would naturally impact their perception that incentives are not effective for alleviating SDOH, as $70 will not cover a substantial amount of an individual’s needs. CBOs that offered nonfinancial incentives, such as food baskets or other items, did so either by including funding for these items in their KIU! budget, through additional grants (as was the case with the CBO that provided free STI/HIV testing), or through in-kind donations. Thus, some CBOs had greater access to additional resources which enabled them to expand beyond what others were able to do based purely on funding from KIU!. Future research examining how high an incentive needs to be effective in increasing recruitment, engagement, and retention should be carried out in future research within HIV prevention to inform incentive implementation. Similarly, future research should examine and compare CBO staff and clients’ perspectives on this concept of incentives potentially being used to alleviate SDOH.

CBO staff also highlighted the importance of timing for financial incentives. Several CBO staff felt that incentives work “in the moment” but that participants would lose interest in the incentive if it were divided across multiple modules into relatively small amounts. Prior research has found that timing of incentives does matter, with incentives being provided “in the moment” of intervention milestone completion being more effective and acceptable than a scenario in which participants must wait to receive the incentive [62]. While our analysis details that CBO staff were largely excited to provide financial incentives and found them beneficial, future research should examine the amount, timing, and type of incentive needed to impact patient behavior, particularly as this relates to CBO’s goals of increasing recruitment, engagement, *and* retention.

Future researchers should also attend to policy determinants of financial incentives. State law varies regarding the legality of incentivizing health promotion and prevention. Arkansas, for example, prohibits the provision of incentives for substance use treatment [63]. In comparison, California allows Medi-Cal contracting authorities to provide nonfinancial incentives for health promotion [63]. However, such incentives must be approved by Medi-Cal. Further, federal law remains blurry on the legality of incentivization. The Department of Health and Human Service’s Office of the Inspector General (OIG) has stated that those providing incentives to patients on Medicare or Medicaid may be committing “wrongful acts” and as a result may be pursued for civil monetary payments [64]. However, the OIG has also stated that “this does not mean that all cash or cash-equivalent payments are unlawful, but that they are subject to case-by-case analysis” [64]. The Substance Abuse and Mental Health Services Administration (SAMHSA) limits contingency management to $75 per client and financial incentives to $30 [65]. Thus, researchers attending to the effectiveness of financial incentives for HIV prevention should also consider state and federal policy as important context for real world implementation.

Incentives for participation in interventions, as opposed to incentives for completing research-related activities (e.g., surveys, interviews), can raise some concerns regarding the validity of implementation outcomes. In this trial, reach of the intervention was a primary outcome; incentives are likely to have affected reach rates as they could have motivated participants to consent to the study and/or to use KIU! As this was a pragmatic trial, the research team desired that CBOs follow typical processes and strategies. As incentives for HIV preventive interventions were commonplace in participating CBOs, incentives were not only permitted but encouraged in the trial to provide future adopting CBOs with estimates of program enrollment and completion that would be obtained when incentives are used. In implementation studies where intervention participation is not commonly incentivized, doing so for all (or some) sites could bias reach rates and inflate the expected number of participants who would engage in the intervention were it to become standard of care. This potential source of bias in implementation research deserves greater attention, particularly in the context of pragmatic implementation trials.

These findings should be considered with the following limitations. First, the aim of the KIU! trial was not to assess the effectiveness of incentives. CBOs were able to choose whether to include incentives as part of their implementation, but they were not required to, nor was the trial designed to quantitatively assess the effectiveness of incentives across time. Second, incentives only comprised a small portion of interviews with CBO staff. Interviews primarily focused on barriers and facilitators to implementing KIU!. Despite these limitations, these findings provide formative findings for future research to attend to the effectiveness and implementation of financial incentives for HIV prevention DHIs.

## Conclusion

Our analysis is among the first to examine CBO staff perceptions of implementing financial incentives for a digital HIV prevention intervention in the U.S. Our analysis details that staff at HIV-focused CBOs find incentives important and needed, particularly to attend to SDOH. However, staff members highlighted the need to significantly increase incentive amounts for them to be impactful on recruitment and retention. Future research is needed to assess the effectiveness of financial incentivization of digital HIV prevention interventions and to identify the amount an incentive should be to increase recruitment and retention.

## Figures and Tables

**Table 1:** Interviewees’ roles and length in roles

CBO	Staff 1		Staff 2	
		
	Position	Length in Role	Position	Length in Role

01	Program Supervisor	0–5 years	Peer Health Navigator	0–5 years
02	Special Projects Lead	0–5 years	Prevention Programs Lead	0–5 years
03	Prevention Navigator	0–5 years	--	--
04	Supervisor of Prevention	0–5 years	--	--
05	Outreach Worker	0–5 years	Prevention Manager	0–5 years
06	HIV Program Manager	0–5 years	--	--
07	HIV Peer Advocate	0–5 years	HIV Services Manager	0–5 years
08	Director of HIV and STI Prevention	0–5 years	HIV and STI Prevention Coordinator	0–5 years
09	Tester & Counselor	0–5 years	--	--
10	Assistant Director of Health Services	0–5 years	--	--
11	Infectious Disease Program Coordinator	11–15 years	PrEP Navigator	0–5 years
12	Outreach & Testing Coordinator	0–5 years	--	--
13	Director of Health Equity	Unclear	--	--
14	Programming Director	0–5 years	--	--
15	Ryan White Program Manager	0–5 years	Director of Health Equity	0–5 years
16	Coordinator	16 + years	Community Health Educator	0–5 years
17	Outreach Prevention Specialist	0–5 years	--	--

**Table 2:** Total incentive given per CBO across implementation

CBO	Incentives in proposals	Incentives as of October 2020	Incentives as of September 2021

1	0	20	50
2	100	100	100
3	0	0	75
4	95	125	0
5	55	55	0
6	35	35	35
7	35	45	45
8	40	0	100
9	5	0	30
10	30	20	125
11	10	40	35
12	0	100	100
13	0	30	85
14	75	0	175
15	100	150	100
16	10	40	125
17	40	40	55
18	40	40	60
19	80	0	0
20	60	60	60
21	20	0	100
22	40	0	50

Due to the limitations of data collection, these numbers are not cumulative. Rather, they are one-time reports by CBOs that do not reflect the stagnancy or variation of these amounts between proposal and October 2020 or between October 2020 and September 2021
